# What explains health in persons with visual impairment?

**DOI:** 10.1186/1477-7525-12-65

**Published:** 2014-05-03

**Authors:** Juliane Leissner, Michaela Coenen, Stephan Froehlich, Danny Loyola, Alarcos Cieza

**Affiliations:** 1 Department of Medical Informatics, Biometry and Epidemiology – IBE, Chair for Public Health and Health Services Research, Research Unit for Biopsychosocial Health, Ludwig-Maximilians-University (LMU) Munich, Marchioninistr 17, Munich 81377, Germany; 2 ICF Research Branch in cooperation with the WHO Collaborating Centre for the Family of International Classifications in Germany (at DIMDI), Nottwil, Switzerland; 3 ARIS MVZ Nuernberg, Neumeyerstr 48, Nuernberg 90411, Germany; 4 Faculty of Social and Human Sciences, School of Psychology, University of Southampton, Highfield Campus, Southampton SO17 1BJ, United Kingdom; 5 Swiss Paraplegic Research, Guido-Zäch-Str. 4, Nottwil 6207, Switzerland

**Keywords:** Visual impairment, Functioning, Health, International Classification of Functioning, Disability and Health, Quality of life

## Abstract

**Background:**

Visual impairment is associated with important limitations in functioning. The International Classification of Functioning, Disability and Health (ICF) adopted by the World Health Organisation (WHO) relies on a globally accepted framework for classifying problems in functioning and the influence of contextual factors. Its comprehensive perspective, including biological, individual and social aspects of health, enables the ICF to describe the whole health experience of persons with visual impairment. The objectives of this study are (1) to analyze whether the ICF can be used to comprehensively describe the problems in functioning of persons with visual impairment and the environmental factors that influence their lives and (2) to select the ICF categories that best capture self-perceived health of persons with visual impairment.

**Methods:**

Data from 105 persons with visual impairment were collected, including socio-demographic data, vision-related data, the Extended ICF Checklist and the visual analogue scale of the EuroQoL-5D, to assess self-perceived health. Descriptive statistics and a Group Lasso regression were performed. The main outcome measures were functioning defined as impairments in Body functions and Body structures, limitations in Activities and restrictions in Participation, influencing Environmental factors and self-perceived health.

**Results:**

In total, 120 ICF categories covering a broad range of Body functions, Body structures, aspects of Activities and Participation and Environmental factors were identified. Thirteen ICF categories that best capture self-perceived health were selected based on the Group Lasso regression. While Activities-and-Participation categories were selected most frequently, the greatest impact on self-perceived health was found in Body-functions categories. The ICF can be used as a framework to comprehensively describe the problems of persons with visual impairment and the Environmental factors which influence their lives.

**Conclusions:**

There are plenty of ICF categories, Environmental-factors categories in particular, which are relevant to persons with visual impairment, but have hardly ever been taken into consideration in literature and visual impairment-specific patient-reported outcome measures.

## Background

Visual impairment (VI) is defined as blindness or low vision [1] and is associated with important limitations in functioning [2,3]. Psychological distress, difficulties in activities of daily living (ADL) and low health-related quality of life have consistently been reported in persons with VI (PVI) [4-10]. To assess these limitations comprehensively the patient perspective has to be taken into account. In ophthalmology traditional objective clinical measures, such as best corrected visual acuity (BCVA), are being complemented by the assessment of patients’ perception of their visual function, functioning in general and quality of life [11]. Generic patient-reported outcome measures, such as the Medical Outcome Study Short Form 36 (SF-36) [12], EuroQoL-5D (EQ-5D) [13], utility values, such as the time trade-off and standard gamble, and condition-specific patient-reported outcome measures, like the Visual Function 14-item Scale (VF-14) [14] and the Activities of Daily Vision Scale (ADVS) [15], the Daily Living Tasks Dependent on Vision (DLTV) [16] and the National Eye Institute Visual Function Questionnaire (NEI VFQ) [17], have been used to address functioning and quality of life in PVI [18-28].

There is little standardisation regarding the use of these outcome measures making comparisons among studies difficult. However, for the comparison of study outcomes calculation of effect sizes or structural equation modelling, as well as mapping the outcome measures used in these studies to the International Classification of Functioning, Disability and Health (ICF) [29] can be applied. Studies using patient-reported outcome measures typically only cover selected aspects of the whole experience associated with VI. Generic, as well as vision-specific, health-status measures and health-related quality-of-life instruments also vary considerably regarding the concepts included [30-32]. It is also important to recognise that these instruments have been developed to measure the consequences of VI without sufficiently taking into account the influence of environmental and personal factors as defined by the ICF. However, selected personal and environmental factors (e.g., age, gender, use of assistive devices) have often been assessed as potential confounders in intervention studies focusing on rehabilitation in PVI or in cohort studies [33].

**Figure 1:**
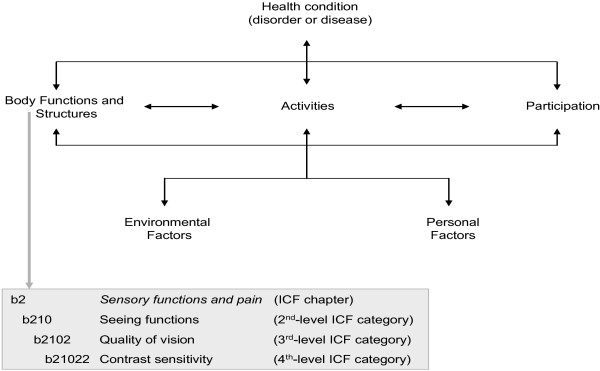
The bio-psycho-social perspective of the ICF and its hierarchical structure.

The ICF adopted by the World Health Organisation (WHO) in 2001 relies on a globally accepted framework for classifying problems in functioning and the influence of contextual factors, such as environmental and personal factors. Its comprehensive perspective, including biological, individual and social aspects of health, enables the ICF to describe the whole health experience of PVI (see Figure 1). The perspective that served as a basis for the development of the ICF rests upon a bio-psycho-social perspective, i.e. it covers functioning and disability with its components Body Functions and Body Structures, Activities and Participation, as well as Personal and Environmental Factors. The classification contains a total of 1424 ICF categories allotted to these components. The component Personal Factors has not yet been classified. According to WHO’s definition ICF categories are “mutually exclusive, i.e. no two categories at the same level share exactly the same attributes” (p. 211; [30]), and organized in a hierarchic structure with up to four levels. However, the mutual exclusivity assumption of some ICF categories is now under discussion [34]. Each category is denoted by a code composed of a letter that refers to the components of the classification (b: Body Functions; s: Body Structures; d: Activities and Participation; e: Environmental Factors) and is followed by a numeric code starting with the chapter number (one digit) and followed by the second level (two digits) and the third and fourth levels (one digit each) of the classification (see Figure 1). A higher-level category shares the attributes of the lower-level category to which it belongs, i.e., the use of a higher-level category (b2102 Quality of vision) automatically implies that the lower-level category is applicable (b210 Seeing functions).

The open question is the extent to which the ICF could be used to comprehensively describe the problems in PVIs’ functioning. It could also help in clinical practice and research to select ICF categories that are most relevant for PVI. Since functioning is the operationalization of health from WHO perspective and in the context of the ICF the subjective perception of PVIs’ health seems to be the most appropriate external standard to perform such a selection. The objectives of this study are, therefore, (1) to analyze whether the ICF can be used to comprehensively describe PVIs’ functioning and the environmental factors that influence their lives and (2) to select the ICF categories that best capture PVIs’ self-perceived health.

## Methods

### Study design

The study was carried out as an empirical cross-sectional study. It received ethics approval from the Ethics Committee of the Ludwig-Maximilian University in Munich (Germany) in accordance with the Declaration of Helsinki and the Amendment of Somerset West (1996).

Although a severe visual impairment of both eyes is referred to as blindness the term is not consistently defined in different countries. The WHO has compiled a comprehensive classification of visual impairment to achieve comparability [35]. However, since the study was performed in Germany the German definition for VI and blindness [36] was taken into account. In this definition blindness and VI is a BCVA of less than 1/50 and a BCVA between 1/50 and 20/63, respectively. As these categories are comparable with the WHO categories data could easily be transformed (Table 1).

**Table 1:** **Definition of VI and blindness according to the World Health Organisation and the International Classification of Disease** (**ICD**-**10**) **currently applied in Germany (based on Snellen charts)**

**WHO category of VI**	**VA with best possible correction**		**ICD-10-GM**
	**Maximum less than**	**Minimum equal to or better than**	
Mild or no VI (0)		20/63	Mild or no VI
Moderate VI (1)	20/63	20/200	Moderate VI
Severe VI (2)	20/200	1/20	Severe VI
Blindness (3)	1/20	1/50	High-grade VI
Blindness (4)	1/50	Light perception	Blindness
Blindness (5)	No light perception	No light perception	Blindness

VI = visual impairment; VA = visual acuity.

### Sample

Patients were included if they (1) were visually impaired according to the International Classification of Disease ICD-10 (H54.0-H54.2), (2) were at least 18 years old, (3) had been informed about the study, (4) had understood the purpose of the study and (5) had signed the informed consent form.

### Measurement instruments

The following measurement instruments were used:

#### Extended ICF Checklist

The Extended ICF Checklist is based upon WHO’s ICF Checklist (Version 2.1a) [37]. The checklist provides a list of 128 first- (n = 5) and second-level (n = 123) ICF categories aiming to assess and record information on functioning (e.g., energy and drive functions, writing, participation in social activities), as well as relevant environmental factors (e.g., assistive devices). When completing the checklist all information available should be used by the health professionals assessing the data (e.g., written records, direct observation and respondent). In our study the assessment of the checklist was mainly based on the information retrieved from one-to-one interviews of the health professional and the respective study participants (see Data collection).

For this study ICF categories originally not included in this ICF Checklist were added. The inclusion of these additional categories was based on commonly used VI-specific patient-reported outcome measures (VF-14, VFQ-25, DLTV, ADVS) whose items had been linked to the ICF, as well as expert opinion in the field of VI. This resulted in the Extended ICF Checklist covering a broader spectrum of possible relevant health areas for individuals with VI. The Extended ICF Checklist includes 217 categories. Sixty-three second-level, 25 third- and four fourth-level categories were added to the original ICF Checklist. Three first-level categories from the original ICF Checklist were excluded because they were covered by second-level categories added to the original ICF Checklist.

The qualifier scale to quantify the degree of patients’ problems in each of these categories was: 0 = no problem, 1 = mild problem, 2 = moderate problem, 3 = severe problem, 4 = complete problem, 8 = not specified (the available information is not sufficient to quantify the severity of the problem), 9 = not applicable (e.g., the category d760 Family relationships is not applicable to a patient without a family). Environmental factors were quantified with a five-point qualifier scale that denotes the extent to which an environmental factor functions as a barrier (1 = mild barrier, 2 = moderate barrier, 3 = severe barrier, 4 = complete barrier) or a facilitator (+1 = mild facilitator, +2 = moderate facilitator, +3 = severe facilitator, +4 = complete facilitator).

#### EuroQol-5D – Visual analogue scale (VAS)

The EQ-5D 20-cm vertical VAS from 0 to 100 was used to measure self-rated health. Its endpoints are labelled ‘Best imaginable health state’ (100) and ‘Worst imaginable health state’ (0). The following written instruction is given to the respondents: “To help people say how good or bad a health state is, we have drawn a scale (rather like a thermometer) on which the best state you can imagine is marked 100 and the worst state you can imagine is marked 0. We would like you to indicate on this scale how good or bad your own health is today, in your opinion. Please do this by drawing a line from the box below to whichever point on the scale indicates how good or bad your health state is today.” The EQ-5D and its VAS is proven to be a reliable and valid measure in a variety of clinical populations likewise in vision [38]. Besides its use in health-economic studies, the EQ-5D VAS has often been used as a single-time measure to assess health-related quality of life in studies using a cross-sectional study design [39,40].

### Data collection

A convenient sample of patients was recruited in the Eye Clinic of the Ludwig-Maximilian-University, Munich (Germany) and a registered association for PVI in Munich (“Bayerischer Blinden- und Sehbehindertenverein”). Data were collected by two researchers with medical background (JL: senior medical student, DL: dentist; each assessing half of the recruited patients) based on (1) patient records including VI-related and socio-demographic data and (2) one-to-one interviews assessing the Extended ICF Checklist described above. Data collection was carried out in a quiet room and lasted approximately one hour. After the interview patients were asked whether other important issues should have been discussed and additional ones were documented. Patients filled in the EQ-5D VAS before or after the interview. Those with severe VI were helped by the interviewer or a patient proxy.

### Data analysis

#### Descriptive analysis of the study population

Descriptive statistics of socio-demographic and VI-related data were performed to characterize the sample. Analyses were stratified by VI into four categories (moderate, severe, higher-grade VI and blindness) according to the German definition of VI and blindness (see Table 1).

#### Description of functioning and environmental factors

Descriptive statistics were performed to identify the ICF categories that describe PVIs’ problems of functioning and the environmental factors that influence their lives. ICF categories qualified as ‘not specified’ (8) were recoded as missing data, whereas categories coded as ‘not applicable’ (9) were recoded as 0 (not impaired, limited or restricted). Third- and fourth-level ICF categories were represented by their respective second-level categories to ensure comprehensibility. ICF categories of the components Body Functions, Body Structures and Activities and Participation that were impaired, limited or restricted (qualified as 1 to 4) by more than five percent of the participants were reported. This arbitrary cut-off was applied to facilitate the reading of the results section. Environmental-factors categories were divided into barriers and facilitators. A cut-off for facilitators was not applied, as all categories were reported in more than five percent of the study participants. Results were stratified by VI into four categories as indicated above (see Table 1).

Additional important issues mentioned by the participants after the interview were linked to ICF categories in a systematic and standardised way based on established linking rules [41,42]. According to these linking rules each issue was linked to the ICF category representing this issue most precisely. If a concept described an aspect which is not covered by the ICF, the code ‘not covered’ (nc) was attributed (e.g., time-related aspects, overall quality of life). Issues identified as Personal factors (e.g., coping with the health condition) were documented as ‘pf’.

#### Selection of ICF categories that best capture different levels of self-perceived health

Group Lasso regression analysis was performed to select the ICF categories that best capture self-perceived health in PVI [43,44]. The EQ-5D VAS was used as dependent variable to address self-perceived health. The ICF categories of the Extended ICF Checklist (reported as a problem for more than 5% of the patients) addressing aspects of functioning and disability, as well as environmental factors, were used as independent variables. Age, gender and time since diagnosis were controlled for in the model.

The EQ-5D VAS has recently been applied as dependent variable in regression analyses in several studies covering a broad range of settings [45-48]. The advantage of using the EQ-5D VAS as dependent variable is that it provides a quantitative (metric) measure of general health judged by the respondents. In contrast, other health-related quality of life outcome measures (e.g., SF-36) include items explicitly addressing aspects of functioning and disability as defined by the ICF (e.g., feeling depressed or anxious, pain, limitation in vigorous activities) [30]. Therefore, these measures are not appropriate to be used as dependent variables when examining the effect of functioning on general health.

Group Lasso is a regression technique that, in addition to the estimation of regression coefficients, allows for the selection of dummy coded categorical independent variables (e.g., ICF categories) that best explain the variance of a dependent variable [49]. Thus, all response options of the ICF categories, even the negative values of the environmental factors (barriers), are treated as dummy coded variables with “no problem” serving as the reference response option. Therefore, there is no need of additional transformations of the available data (e.g., dichotomizing ICF categories into 0 = no problem and 1 = problem without further differentiating the degree of the problem). In addition, the ordinal scale level of independent variables can be taken into account. Finally, Group Lasso regression can be used when the number of regression coefficients that must be estimated is large or even exceeds the sample size [43].

To obtain the best (or final) model, the size of a so-called penalty parameter must be defined. If the penalty is 0 all independent variables are included in the model with non-zero regression coefficients. With increasing penalty, more regression coefficients are estimated to be zero, i.e. less independent variables are included in the model. Finally, for a very large penalty, only the intercept and possible forced-in variables remain in the model. The optimal size of the penalty is defined as the penalty that minimizes the mean-squared prediction error (i.e. the squared difference between the observed and the predicted value of the dependent variable) in 5-fold cross-validation (i.e. the data is randomly split into 5 approximately equal sized parts and then the model is successively estimated based on four fifth of the data and validated on the remaining fifth). Finally, the model is re-estimated on the complete dataset using the identified optimal penalty. Because of this model selection strategy, model selection in Group Lasso regression does not rely on p-values or statistical significance. The independent variables with non-zero regression coefficients are considered relevant, while the others are considered not relevant (and have regression coefficients of zero). Therefore, p-values cannot be obtained based on this method. Furthermore, concerns regarding multiple testing are not applicable, as no statistical test is performed.

Descriptive data analysis and Group Lasso regression were performed by using SPSS Statistics v17.0 (SPSS Inc., Chicago, IL, USA) and R 2.13.0 (R Foundation for Statistical Computing, Vienna, Austria), respectively.

## Results

### Descriptive analysis of the study population

In total, 105 PVI (n = 66 females, 62.9%) with a mean age at interview of 63.3 years (±18.8) ranging from 25 to 93 were included. The mean time since diagnosis of VI was 16.8 years (±17.8). Fifty-four participants (51.4%) reported having had their vision affected for ten years or longer and 16 participants (15.2%) since birth. Additional socio-demographic and VI-related data, as well as the EQ-5D VAS data, are listed in Table 2. It is conspicuous that the mean age of study participants in the blind group is considerably lower compared to the other groups. Mean of the EQ-5D VAS (0 – 100) of the entire sample is 58.9 which is considerably lower than the mean of the German general population (M = 82.2) [50].

**Table 2:** **Socio**-**demographic and VI**-**related characteristics of the participants (N** = **105)**

**Characteristics**	**Total**	Category of VI*			
		**Moderate**	**Severe**	**High**-**grade**	**Blindness**
Number of PVI, n (%)	105 (100.0)	40 (38.1)	25 (23.8)	14 (13.3)	26 (24.8)
Age; years, mean (SD)	63.3 (18.8)	71.4 (15.6)	63.6 (16.4)	71.4 (15.7)	46.4 (16.6)
Time since diagnosis; years, mean (SD)	16.8 (17.8)	8.2 (12.7)	16.9 (17.3)	15.1 (19.5)	31.0 (16.3)
Gender; female, n (%)	66 (62.9)	27 (67.5)	14 (56.0)	8 (57.1)	17 (65.4)
EQ-5D VAS; mean (SD)	58.9 (22.5)	56.2 (21.2)	58.0 (19.5)	46.4 (24.8)	70.7 (22.0)

VI = visual impairment; PVI = persons with visual impairment; EQ-5D VAS = EuroQoL-5D visual analogue scale.

*Moderate VI: BCVA 20/63 - 20/200, Severe VI: BCVA 20/200 - 1/20, High-grade VI: BCVA 1/20 - 1/50, Blindness: BCVA ≤ 1/50.

### Description of functioning and environmental factors

Of the 188 first- and second-level ICF categories of the Extended ICF Checklist 129 categories (68.6%) were relevant in PVI applying the 5% cut-off. Thus, 23 categories in Body Functions, 2 in Body Structures, 63 in Activities and Participation and 41 in Environmental Factors were identified. Absolute and relative frequencies of the identified ICF categories for the entire sample and stratified by VI are shown in Tables 3, 4 and 5.

**Table 3:** ICF categories referring to Body functions and Body structures

**ICF code**	**ICF category title**	**Total sample (N = 105)**						**Sample stratified by VI-category**^**#**^			
		**ICF qualifier (1–4)**^**§**^						**Moderate (n = 40)**	**Severe (n = 25)**	**High-grade (n = 14)**	**Blindness (n = 26)**
		**1**	**2**	**3**	**4**	**Sum 1-4**		**Sum 1-4**			
		**n**	**n**	**n**	**n**	**n**	**%**	**%**	**%**	**%**	**%**
b210	Seeing functions	4	18	61	22	105	100.0	100.0	100.0	100.0	100.0
b220	Sensations associated with the eye and adjoining structures	46	15	4	1	66	62.9	70.0	72.0	64.3	42.3
b126	Temperament and personality functions	30	25	6		61	58.1	62.5	68.0	42.8	50.0
b130	Energy and drive functions	34	12	5		51	48.6	65.0	44.0	35.7	34.6
b215	Functions of structures adjoining the eye	21	12	3	1	37	35.6	27.5	44.0	28.6	44.0
b280	Sensation of pain	21	9	5		35	33.3	40.0	24.0	28.6	34.6
b152	Emotional functions	20	11			31	29.8	37.5	24.0	23.1	26.9
b134	Sleep functions	16	9	5		30	28.8	32.5	25.0	28.6	26.9
b235	Vestibular functions	16	7			23	21.9	15.0	40.0	14.3	19.2
b240	Sensations associated with hearing and vestibular function	15	5			20	19.2	12.8	28.0	21.4	19.2
b144	Memory functions	19	1			20	19.0	25.0	20.0	14.3	11.5

ICF categories are reported that were coded as at least mildly impaired in more than five percent of participants, ordered by frequency (n). Results are shown for total sample and stratified by VI-category.

VI = visual impairment.

^§^1: mild difficulty, 2: moderate difficulty, 3: severe difficulty, 4: complete difficulty.

^#^Moderate VI: BCVA 20/63 - 20/200, Severe VI: BCVA 20/200 - 1/20, High-grade VI: BCVA 1/20 - 1/50, Blindness: BCVA ≤ 1/50.

**Table 4:** ICF categories referring to activities and participation

**ICF code**	**ICF category title**	**Total sample (N=105)**						**Sample stratified by VI-category**^**#**^			
		**ICF qualifier (1-4)**^**§**^						**Moderate (n=40)**	**Severe (n=25)**	**High-grade (n=14)**	**Blindness (n=26)**
		**1**	**2**	**3**	**4**	**Sum 1-4**		**Sum 1-4**			
		**n**	**n**	**n**	**n**	**n**	**%**	**%**	**%**	**%**	**%**
d325	Communicating with - receiving - written messages	23	42	31	5	101	97.1	97.4	100.0	100.0	92.3
d345	Writing messages	24	44	29	3	100	95.2	97.5	100.0	100.0	84.6
d170	Writing	20	54	24	2	100	95.2	97.5	100.0	100.0	84.6
d110	Watching	18	26	37	16	97	92.4	85.0	96.0	100.0	96.2
d475	Driving	5	3	26	61	95	91.3	90.0	92.0	92.3	92.3
d460	Moving around in different locations	46	33	13	2	94	91.3	85.0	95.8	92.3	96.2
d166	Reading	13	38	30	6	87	82.9	87.5	88.0	92.9	75.4
d650	Caring for household objects	31	18	30	7	86	82.7	80.0	72.0	100.0	88.5
d920	Recreation and leisure	34	30	19	2	85	82.5	82.5	84.0	83.3	80.8
d620	Acquisition of goods and services	26	32	23	2	83	79.8	67.5	76.0	100.0	92.3
d315	Communicating with - receiving - nonverbal messages	24	26	17	16	83	79.8	60.0	83.3	92.9	100.0
d470	Using transportation	39	34	7	2	82	78.8	72.5	88.0	92.3	73.1
d455	Moving around	25	32	19	1	77	74.8	72.5	70.8	69.2	84.6
d865	Complex economic transactions	41	24	7	5	77	74.0	70.0	64.0	76.9	88.5
d220	Undertaking multiple tasks	32	31	10		73	69.5	65.0	64.0	85.7	73.1
d440	Fine hand use	39	20	8	1	68	64.8	82.5	68.0	71.4	30.8
d640	Doing housework	44	14	8		66	63.5	52.5	56.0	100.0	69.2
d810	Informal education	43	20	2		65	64.4	57.9	66.7	76.9	65.4
d860	Basic economic transactions	32	22	8	2	64	61.5	70.0	56.0	84.6	42.3
d630	Preparing meals	42	14	2	3	61	58.7	42.5	56.0	76.9	76.9
d155	Acquiring skills	41	15	4		60	57.1	57.5	64.0	71.6	42.3
d360	Using communication devices and techniques	45	10	1		56	53.8	61.5	68.0	64.3	23.1
d450	Walking	35	18	2		55	53.4	47.5	70.8	53.8	46.2
d240	Handling stress and other psychological demands	30	17	5		52	50.0	48.7	44.0	71.4	46.2
d230	Carrying out daily routine	26	12	5		43	41.0	42.5	40.0	42.9	38.5
d660	Assisting others	25	8	6	3	42	41.2	28.2	54.2	61.5	38.5
d910	Community life	30	6	6		42	40.8	37.5	40.0	50.0	42.3
d210	Undertaking a single task	30	7	4		41	39.0	25.0	40.0	50.0	53.8
d430	Lifting and carrying objects	24	12	1		37	35.2	37.5	28.0	57.1	26.9
d845	Acquiring, keeping and terminating a job	12	15	7	1	35	33.7	20.0	36.0	7.7	65.4
d140	Learning to read	25	8	1		34	32.4	35.0	48.0	21.4	19.2
d145	Learning to write	22	8	1		31	29.5	30.0	44.0	21.4	19.2
d720	Complex interpersonal interactions	24	4	2		30	28.8	25.0	36.0	30.8	26.9
d830	Higher education	12	14	4		30	28.8	15.0	32.0	15.4	53.8
d520	Caring for body parts	22	5	2		29	27.9	22.5	32.0	53.8	19.2
d175	Solving problems	23	5	1		29	27.6	30.0	24.0	35.7	23.1
d730	Relating with strangers	18	9	1		28	26.9	30.0	28.0	15.4	26.9
d850	Remunerative employment	10	9	7	2	28	26.9	15.0	32.0	7.7	50.0
d550	Eating	19	7		1	27	26.0	7.5	28.0	53.8	38.5
d825	Vocational training	12	12	3		27	26.0	12.5	28.0	15.4	50.0
d540	Dressing	22	4			26	25.2	12.5	33.3	46.2	26.9
d770	Intimate relationships	11	10	3	1	25	24.8	10.5	28.0	25.0	42.3
d560	Drinking	20	4	1		25	24.0	17.5	32.0	38.5	19.2
d750	Informal social relationships	20	3	2		25	24.0	20.0	28.0	23.1	26.9
d465	Moving around using equipment	18	2		2	22	21.8	17.9	16.7	23.1	32.0
d740	Formal relations	14	5	1		20	19.4	17.9	32.0	7.7	15.4
d710	Basic interpersonal interactions	14	4	1		19	18.3	10.0	36.0	23.1	11.5
d335	Producing nonverbal messages	16	2			18	17.1	12.5	4.0	7.1	42.3
d940	Human rights	13	3	1		17	16.3	7.5	20.0	7.7	30.8
d177	Making decisions	11	6			17	16.2	12.5	12.0	35.7	15.4
d320	Communicating with - receiving - formal sign language messages	2	5	3	5	15	14.4	12.5	16.0	14.3	16.0
d820	School education	8	6			14	13.6	5.1	12.0	0.0	34.6
d760	Family relations	10	3		1	14	13.5	12.5	12.0	0.0	23.1
d115	Listening	10	2	1		13	12.4	17.5	16.0	14.3	0.0
d172	Calculating	9	3			12	11.4	5.0	16.0	28.6	7.7
d150	Learning to calculate	9	2			11	10.6	7.7	16.0	14.3	7.7
d355	Discussion	8	1			9	8.6	12.5	8.0	14.3	0.0
d530	Toileting	7	1			8	7.7	7.5	8.0	23.1	0.0
d445	Hand and arm use	4	3			7	6.7	12.5	4.0	7.1	0.0
d950	Political life and citizenship	4	2	1		7	6.7	10.0	0.0	7.7	7.7
d570	Looking after ones health	4	1	1		6	5.8	5.0	4.0	7.7	7.7
d930	Religion and spirituality	3	3			6	5.8	12.5	4.0	0.0	0.0
d120	Other purposeful sensing	6				6	5.7	7.5	8.0	0.0	3.8

ICF categories are reported that were coded as at least mildly limited or restricted in more than five percent of participants, ordered by frequency (n). Results are shown for total sample and stratified by VI-category.

VI = visual impairment.

^§^1: mild difficulty, 2: moderate difficulty, 3: severe difficulty, 4: complete difficulty.

^#^Moderate VI: BCVA 20/63 - 20/200, Severe VI: BCVA 20/200 - 1/20, High-grade VI: BCVA 1/20 - 1/50, Blindness: BCVA ≤ 1/50.

**Table 5:** ICF categories referring to environmental factors

**ICF code**	**ICF category title**	**Total sample(N = 105)**				**Sample stratified by VI-category**^**#**^			
		**Barrier**^**§**^	**Facilitator**^**§t**^			**Moderate (n = 40)**	**Severe (n = 25)**	**High-grade (n = 14)**	**Blindness (n = 26)**
		**Sum 1-4**	**Sum 1-4**	**Sum 1-4**		**Sum 1-4**			
		**n**	**n**	**n**	**%**	**%**	**%**	**%**	**%**
e125	Products and technology for communication	4	94	98	95.1	90.0	100.0	100.0	96.2
e540	Transportation services, systems and policies	14	79	93	90.3	85.0	91.7	84.6	100.0
e240	Light	36	57	93	89.4	90.0	96.0	100.0	76.9
e355	Health professionals	9	84	93	89.4	100.0	80.0	100.0	76.9
e410	Individual attitudes of immediate family members	6	87	93	89.4	90.0	88.0	76.9	96.2
e580	Health services, systems and policies	34	58	92	89.3	82.1	92.0	92.3	96.2
e450	Individual attitudes of health professionals	12	77	89	85.6	90.0	80.0	76.9	88.5
e130	Products and technology for education	2	86	88	85.4	75.0	87.5	84.6	100.0
e310	Immediate family	2	86	88	84.6	90.0	76.0	84.6	84.6
e420	Individual attitudes of friends	4	80	84	80.8	77.5	72.0	69.2	100.0
e460	Societal attitudes	29	53	82	78.8	70.0	68.0	92.3	96.2
e220	Flora and fauna	1	76	77	74.0	72.5	72.0	53.8	88.5
e320	Friends	1	74	75	72.1	62.5	68.0	76.9	88.5
e150	Design, construction and building products and technology of buildings for public use	43	30	73	71.6	66.7	66.7	61.5	88.5
e425	Individual attitudes of acquaintances, peers, colleagues, neighbours and community members	12	61	73	70.2	67.5	64.0	61.5	84.6
e115	Products and technology for personal use in daily living	3	66	69	67.0	60.0	54.2	61.5	88.5
e120	Products and technology for personal indoor and outdoor mobility and transportation	3	66	69	67.0	60.0	58.3	53.8	92.3
e155	Design, construction and building products and technology of buildings for private use	31	36	67	64.4	60.0	52.0	61.5	84.6
e585	Education and training services, systems and policies	31	36	67	64.4	15.4	29.2	30.8	76.9
e325	Acquaintances, peers, colleagues, neighbours and community members	3	63	66	63.5	67.5	44.0	53.8	80.8
e250	Sound	47	18	65	63.1	50.0	66.7	53.8	84.6
e570	Social security services, systems and policies	26	39	65	62.5	60.0	68.0	53.8	65.4
e535	Communication services, systems and policies	9	55	64	62.1	50.0	70.8	53.8	76.9
e560	Media services, systems and policies	8	56	64	62.1	52.5	75.0	53.8	69.2
e415	Individual attitudes of extended family members	10	53	63	60.6	57.5	68.0	69.2	53.8
e225	Climate	42	19	61	58.7	65.0	56.0	53.8	53.8
e110	Products or substances for personal consumption	8	52	60	58.3	52.5	50.0	53.8	76.9
e575	General social support services, systems and policies	23	34	57	54.8	47.5	56.0	46.2	69.2
e465	Social norms, practices and ideologies	24	27	51	49.0	37.5	48.0	53.8	65.4
e315	Extended family	7	41	48	46.2	45.0	52.0	38.5	46.2
e140	Products and technology for culture, recreation and sport		47	47	45.6	45.0	33.3	53.8	53.8
e590	Labour and employment services, systems and policies	25	16	41	41.0	20.5	41.7	30.8	79.2
e455	Individual attitudes of health-related professionals	3	31	34	33.0	22.5	25.0	53.8	46.2
e430	Individual attitudes of people in positions of authority	14	18	32	30.8	20.0	36.0	23.1	46.2
e135	Products and technology for employment		30	30	29.1	20.0	16.7	15.4	61.5
e330	People in positions of authority	9	17	26	25.0	10.0	28.0	30.8	42.3
e525	Housing services, systems and policies	10	14	24	23.1	15.0	16.0	15.4	46.2
e360	Other professionals	3	20	23	22.3	12.5	16.7	30.8	38.5
e340	Personal care providers and personal assistants	2	20	22	21.2	22.5	16.0	15.4	26.9
e550	Legal services, systems and policies	6	12	18	17.5	7.5	16.7	30.8	26.9
e440	Individual attitudes of personal care providers and personal assistants	2	16	18	17.3	20.0	12.0	0.0	26.9

ICF categories are reported that were coded as at least a mild barrier or facilitator in more than five percent of participants, ordered by frequency (n). Results are shown for total sample and stratified by VI-category.

VI = visual impairment.

^§^1: mild facilitator/barrier, 2: moderate facilitator/barrier, 3: substantial facilitator/barrier, 4: complete facilitator/barrier.

^#^Moderate VI: BCVA 20/63 - 20/200, Severe VI: BCVA 20/200 - 1/20, High-grade VI: BCVA 1/20 - 1/50, Blindness: BCVA ≤ 1/50.

The most frequently identified Body-functions categories impaired in PVI were mainly from the chapters b1 Mental functions (e.g., b126 Temperament and personality functions, b130 Energy and drive functions) and b2 Sensory functions and pain (e.g., b210 Seeing functions, b215 Functions of structures adjoining the eye, b220 Sensation associated with the eye and adjoining structures, b280 Sensation of pain). The categories s220 Structures of eyeball and s230 Structures around the eye were the identified categories in the component Body Structures (Table 3). In the component Activities and Participation the 63 ICF categories that were identified as limited or restricted are from all nine ICF chapters ranging from d1 Learning and applying knowledge (11 categories) to d9 Community, social and civic life (5 categories) (Table 4). In the component Environmental Factors all 41 categories were reported as barriers or facilitators by more than 17% of study participants. Categories were distributed among all five chapters: e1 Products and technology (9 categories), e2 Natural environment and human-made changes to environment (4 categories), e3 Support and relationships (8 categories), e4 Attitudes (10 categories) and e5 Services, systems and policies (10 categories) (Table 5).

Additional important issues not addressed in the Extended ICF Checklist and mentioned after the interview were identified in 42 participants (40%). Most of these issues were linked to ICF categories which were more specific than the ICF categories included in the Extended ICF Checklist. However, these categories were addressed by second-level categories included in the Extended ICF Checklist (e.g., ‘Travelling, photography or doing crosswords’ linked to d9204 Hobbies addressed by d920 Recreation and leisure; ‘Lighted magnifier’ linked to e1251 Assistive products and technology for communication addressed by e125 Products and technology for communication). One ICF category, namely e350 Domesticated animals (guide dogs, as well as pets), which was not included in the Extended ICF Checklist, was identified as a facilitator by some participants (n = 5). Some of the issues mentioned by the participants after the interview which were not included in the Extended ICF Checklist relate to Personal Factors. For example, some study participants reported that their personality improved after disease onset. Finally, only one issue which was coded as not covered by the ICF (‘nc’) was identified, namely ‘Needing more time to accomplish daily activities’.

### Selection of ICF categories that best capture different levels of self-perceived health

All ICF categories being a problem for at least 5% of the PVI (n = 129; see Tables 3, 4 and 5) were entered in the Group Lasso regression. Of these, 13 ICF categories were selected that best capture different levels of PVIs’ self-perceived health. The majority of these categories derived from the component Activities and Participation (n = 7). Two and four ICF categories from the components Body Functions and Environmental Factors and none of the Body-Structures categories were selected. The selected ICF categories along with their regression coefficients (beta estimates) are presented in Table 6. These parameters indicate the effect of a certain response to a specific ICF category on expected PVIs’ self-perceived health. To give an example: a person with complete problems in Sensations associated with the eye and adjoining structures is expected to have 10.67 points less in self-perceived health than a person with no problems in this ICF category when controlling for all the other variables in the model. ICF categories not selected in the Group Lasso regression all have regression coefficients of zero.

**Table 6:** Results of the Group Lasso regression

**Variables of the model**		**Beta estimator (β) for the qualifier scale**				
		**β**	**β**_**1**_	**β**_**2**_	**β**_**3**_	**β**_**4**_
**Intercept**		79.149				
Age (years)		-0.347				
Gender (female)		-0.839				
Time since diagnosis (years)		0.274				
b126	Temperament and personality functions		-1.839	-4.088	-5.356	^‡^
b220	Sensations associated with the eye and adjoining structures		-0.372	-6.465	-9.249	-10.670
d155	Acquiring skills		-0.051	-0.09	-0.083	^‡^
d220	Undertaking multiple tasks		-0.022	-1.072	-1.399	^‡^
d240	Handling stress and other psychological demands		-0.344	-1.045	-0.987	^‡^
d315	Communicating with - receiving - nonverbal messages		0.053	0.158	0.249	0.302
d620	Acquisition of goods and services		-0.910	-2.112	-2.993	-2.954
d750	Informal social relationships		-3.098	-2.696	-1.981	^‡^
d810	Informal education		-0.472	-0.896	-1.092	^‡^
e125 +^§^	Products and technology for communication		0.106	0.261	0.588	0.686
e125^#^			-0.068	-0.160	-0.160	-0.160
e325 +^§^	Acquaintances, peers, colleagues, neighbours and community members		0.551	1.213	1.282	^‡^
e325^#^			0.067	0.067	0.067	0.067
e415 +^§^	Individual attitudes of extended family members		1.311	2.65	3.008	2.700
e415^#^			-0.991	-0.969	-0.969	-0.969
e425 +^§^	Individual attitudes of acquaintances, peers, colleagues, neighbours and community members		2.161	4.381	5.511	^‡^
e425^#^			-0.657	-1.103	-1.017	-1.017

Variables of the model; selected ICF categories that best capture different levels of self-perceived health of PVI along with their beta estimator (β).

β_1_: beta estimates for ICF qualifier “1” (mild impairment/limitation/restriction/barrier/facilitator).

β_2_: beta estimates for ICF qualifier “2” (moderate impairment/limitation/restriction/barrier/facilitator).

β_3_: beta estimates for ICF qualifier “3” (severe impairment/limitation/restriction/barrier/facilitator).

β_4_: beta estimates for ICF qualifier “4” (complete impairment/limitation/restriction/barrier/facilitator).

^§^Supportive environmental factor = facilitator.

^#^Hindering environmental factor = barrier.

^‡^Empty stratum; beta estimator could not be calculated.

## Discussion

A broad range of Body functions, Body structures, aspects of Activities and Participation and Environmental factors relevant in PVI were identified in this study. It has been shown that the ICF can be used as a framework to comprehensively describe the problems in functioning of PVI and the Environmental factors which influence their every-day lives. A set of 13 ICF categories was selected by using Group Lasso regression that best capture self-perceived health of PVI.

First, we would like to discuss the ICF categories that can be used to describe functioning and environmental factors of PVI. It stands out that the ICF categories identified in this study cover a broad range of functioning and disability and affect nearly every aspect of daily living as has been described in former publications [51,52]. Besides the obvious impairments in seeing and seeing-related functions, b280 Sensation of pain and mental functions, such as b126 Temperament and personality functions and b130 Energy and drive functions, were reported by more than a third of the study population. This is in line with previous findings reporting that psychosocial factors, such as depression and personality, affect PVIs’ performance and quality of life [53-57].

Activities-and-Participation categories that were identified as limited or restricted most commonly address aspects of communication (e.g., d325 Communicating with – receiving – written messages, d345 Writing messages, d170 Writing, d110 Watching, d166 Reading and d315 Communication with – receiving – nonverbal messages). Reading has not only been described as limited in PVI, but has also been used as a measure for functioning and quality of life [58], whereas limitations in writing have seldom explicitly been stressed in the literature even though writing is addressed in several patient-reported outcomes (e.g., functional ability Quality of Vision (faVIQ) [59], Low Vision Quality-of-life Questionnaire (LVQOL) [60], VF-14). Furthermore, activities from the chapters d4 Mobility (e.g., d475 Driving, d460 Moving around in different locations and d470 Using transportation) and d6 Domestic life (e.g., d650 Caring for household objects, d620 Acquisition of goods and services) were identified as limited by more than two thirds of the study participants. These findings are consistent with the literature [61,62] but offer more precise examples of limitations in every-day activities or restrictions in participation.

With this study we also identified several Environmental factors influencing PVIs’ lives. Up to now, there has been very little research on environmental factors and VI. Taking into account that all categories in the Extended ICF Checklist were reported along with the frequencies with which study participants reported them, the lack of research becomes even more apparent. It should be mentioned that PVI reported far more facilitators than barriers. Facilitators, such as e125 Products and technology for communication, e130 Products and technology for education and e115 Products and technology for personal indoor and outdoor mobility and transportation, emphasise the importance of adequate vision aids, magnifiers, big-buttoned telephones, talking clocks, canes [63]. This result also underlines the importance of vision-related technology and assistive devices in the rehabilitation process of PVI. Study participants reported noise to be misleading when participating in traffic, even as a pedestrian and in winter. For instance, snow can present an insurmountable obstacle due to its noise-reducing effect and by blurring existing boarders such as kerbs as highlighted by several study participants. These are just two possible reasons why the categories e250 Sound and e225 Climate constitute two of the three most common barriers reported by the study participants. Categories in chapters e3 Support and relationship, such as e355 Health professionals, e310 Immediate family and e320 Friends, as well as e4 Attitudes of the very same people, were also reported to be facilitators by more than two thirds of the study population. Furthermore, there are plenty of categories that have been reported to be barriers as well as facilitators, like e150 Design, construction and building products and technology of buildings for public use, e155 Design, construction and building products and technology of buildings for private use, e580 Health services, systems and policies or e585 Education and training services, systems and policies. This indicates that public services which are employed to improve every-day lives of visually impaired and blind individuals are underachieving.

The results of our study show that the ICF can be used to comprehensively describe problems in functioning of PVI and environmental factors influencing their lives. About 40 percent of the participants mentioned additional issues after the assessment of the Extended ICF Checklist. However, the majority of these issues were covered by the ICF (third-level categories, Personal factors). There was only one additional category that was labelled as ‘not covered’ by the ICF which referred to time-related aspects (‘Needing more time to accomplish daily activities’).

Second, we like to discuss the selected ICF categories that best capture PVIs’ self-perceived health. When discussing this topic it is important to realize that the ICF categories selected by using Group Lasso regression often do not include the categories that have been selected most commonly as impaired, limited, restricted or as a barrier or facilitator. Since all of our study participants were visually impaired, the categories b210 Seeing functions and d166 Reading, for example, were qualified as severe or complete impaired in all study participants. These categories besides others could not be selected applying regression analysis, as only categories showing variation can explain differences in self-perceived health. However, it is obvious that these aspects of functioning are highly relevant in patients’ every-day lives and as intervention goals in rehabilitation. Applying the Group Lasso regression the majority of the selected categories (n = 13) was derived from the component Activities and Participation. It has been previously reported that VI leads to restrictions in participation [64,65,41,28] which is defined as problems that an individual may experience in his/her involvement in life situations [32]. Activities-and-Participation categories showing the highest values of beta estimators in the Group Lasso regression were d620 Acquisition of goods and services and d750 Informal social relationships. The latter correlates well with the findings in the component Environmental Factors and will thus be discussed later on. It is interesting that the Centre for Eye Research in Australia ranked the ‘Household and Personal Care’ domain low in order of difficulty, acting on the assumption that familiarity with the household environment makes the tasks easier to perform [41]. Existing outcome measures, such as the VFQ-25, include questions on single tasks, e.g., reading small print and going down stairs at night [19], but hardly include items that need a combination of skills. The category d620 Acquisition of goods, which was not only reported as limited by 83 percent of PVI, but also has a high beta estimator, requires a combination of skills, such as reading print and moving around in different locations. It seems that existing outcome measures have not been able to grasp the difficulty of every-day life by keeping the questionnaires short and practical. In accordance with these considerations, d220 Undertaking multiple tasks is also one of the selected ICF categories that best captures PVIs’ self-perceived health.

Although ICF categories from the component Activities and Participation have been selected most frequently, the Body-Functions categories are the ones which have the greatest effect on self-perceived health of PVI, showing the highest beta estimators in the Group Lasso regression. One of these categories is b220 Sensations associated with the eye and adjoining structures that includes sensations of tired, dry and itching eye and related feelings. A complete impairment of this body function results in a possible decrease of more than 11% on the self-perceived health scale of the EQ-5D. However, no literature on this subject can be found. Looking at VI-specific measures, the VFQ-25 includes a question regarding this body function, but the VF-14, the DLTV and the ADVS do not address this subject. These findings indicate that the degree to which sensations associated with the eye are related to PVIs’ self-perceived health has been underestimated or undetected so far. The high beta estimator of category b126 Temperament and personality functions, which includes functions of extra- or introversion, agreeableness, conscientiousness, openness to experience and psychic stability, coincides with current literature.

The Environmental factors selected as facilitators or barriers when explaining self-perceived health in PVI mostly address personal relationships. The Blue Mountain Eye Study showed that visually impaired persons are more likely to use support than persons with good vision [66]. In accordance with these results, we found support of e325 Acquaintances, peers, colleagues, neighbours and community members to be associated with self-perceived health in PVI. It is conspicuous that the latter category is always associated with a positive beta estimate and, therefore, always increases self-perceived health regardless of whether the category has been reported as a barrier or a facilitator. We hypothesize that social interaction as such is more important than the kind of support. Thus, being involved in social interactions with others and getting support from others seem to increase self-perceived health regardless of the quality of these interactions and the appraisal of the received support as hindering or supportive factor.

We want to point out that the mean self-perceived health score of PVI adds up to 59 points, and the subgroup of blind individuals scores about 71 points. This might be due to the fact that study participants of this group were approximately 17 years younger than the entire study population. According to the Group Lasso regression this would account for an increase of 5.8 points on the self-perceived health scale of the EQ-5D. Additionally, blind individuals have been living with their diagnosis for about 14 years longer compared to the total sample of participants, which would cause a further increase of 3.9 points. This, however, does not explain the differences among the subgroups. One possible explanation for this phenomenon might be that most of the blind individuals had coped with their loss of vision over their lifetimes to a greater extent than individuals experiencing progressive visual-functioning problems.

Preliminary work on a ICF-based content comparison of existing vision-related patient-reported outcomes has shown that most of the selected categories, except for b220 Sensations associated with the eye and adjoining structures and e125 Products and technology for communication, that best explain self-perceived health of PVI are not taken into account in commonly used questionnaires (e.g., VF-14, NEI VFQ, DLTV, ADVS) [67]. Che Hamzah and colleagues already published a systematic review on vision instruments mapping these instruments to the components of the ICF. However, a detailed ICF-based analysis is still missing [32]. Thus, it might be worthwhile to examine and compare the content of existing instruments using the ICF as a reference and taking into account the ICF categories selected in this study. Depending on the results of this comparison it possibly might be necessary to re-evaluate some of the questionnaires or even to develop a new ICF-based questionnaire addressing the areas of functioning identified in this study. Massof and colleagues [68,69], and even more consequently Bruijning and colleagues [62,70] already developed an ICF-based instrument, namely the Activity Inventory and the Dutch ICF Activity Inventory, respectively, providing a goal attainment approach for rehabilitation of PVI. Both instruments assess the difficulties of specific tasks – covering the ICF categories of the Activities and Participation component – that belong to goals relevant from the patient perspective.

In addition, the results of this study can be used as part of the revision process of the ICD-11. A newly developed axis called “functioning properties” serves as a link to allow for joint usage of the ICD and the ICF. These functioning properties are proposed to be included in the ICD revision process [71]. Therefore, these results may provide a valuable contribution to pinpointing the most important aspects of functioning in PVI which can be compared to functioning properties used in the revision process of the ICD.

**Figure 2:**
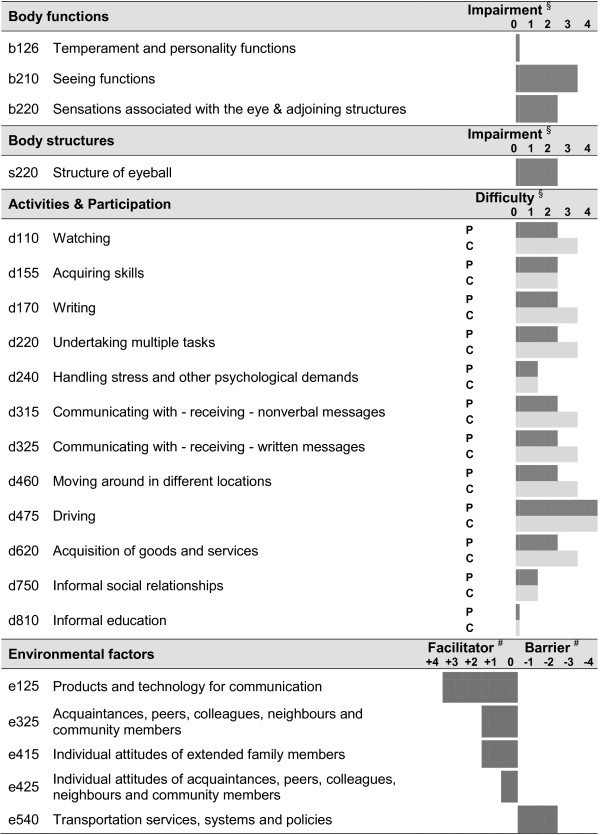
Functioning profile for PVI applying the ICF qualifier.

The results of this study can also be used to create a functioning profile for PVI as shown in Figure 2. It consists of the ICF categories selected in the regression analysis and of ICF categories considered a problem by more than 90 percent of PVI in the descriptive analysis. The categories included in this functioning profile can serve as a checklist for problems PVI may experience in their every-day lives, as well as environmental factors relevant to them. This functioning profile, therefore, provides a useful guide for the planning, follow-up and reporting of health-care interventions [72]. This approach might be seen in line with the perspective of personalized medicine aiming to tailor medical decisions, practices, and/or products to the individual patient.

This study has some limitations which should be mentioned. One limitation is the sample size of 105 patients. However, Gertheiss and colleagues assume that a sample size of 105 participants is sufficient to conduct Group Lasso regression analyses [36]. Nevertheless, the results of this study should be interpreted with caution; we recommend to conduct further studies with larger samples using Group Lasso regression analyses. There was only one study centre located in Germany. Further studies in other countries are needed to validate the results of this investigation. Patients filled in the EQ-5D before or after the interview. We are aware that this could have affected the rating on self-perceived health. Recoding the qualifier “9” (not applicable) to “0” (not impaired, limited or restricted; no facilitator/barrier) might be worthwhile to discuss. We used this proven recoding strategy [45,73] for example for study participants who were unemployed because of their health condition or were (early) retired when coding d850 Remunerative employment.

## Conclusions

The ICF can be used as a framework to comprehensively describe PVIs’ problems and the environmental factors which influence their lives. In light of existing approaches to develop ICF-based outcome measures in the field of VI it would be worthwhile to bring together the results of this study with research already performed in this field. We highly recommend to start with the mapping of existing VI-specific outcome measures to the categories of the ICF to facilitate the comparison of outcome measure used in research and rehabilitation.

## Abbreviations

ADL: Activities of daily living; ADVS: Activities of daily vision scale; BCVA: Best corrected visual acuity; DLTV: Daily living tasks dependent on vision; EQ-5D: EuroQoL 5D; ICF: International Classification of Functioning, Disability and Health; LVQOL: Low Vision Quality-of-life Questionnaire; nc: Not covered; NEI VFQ: National Eye Institute Visual Function Questionnaire; pf: Personal factors; PVI: Persons with visual impairment; QoL: Quality of life; SF-36: Medical Outcome Study Short Form 36; VF-14: Visual function 14-item scale; VI: Visual impairment; WHO: World Health Organisation.

## Competing interests

The authors declare that they have no competing interests.

## Authors’ contributions

JL organized and carried out data collection, performed statistical analyses and drafted the manuscript. MC participated in the design of the study and supervised the statistical analyses as well as the drafting of the manuscript. SF participated in the design of the study and coordination. DL organized and carried out data collection. AC conceived of the study and participated in its design and coordination. All authors read and approved the final manuscript.

## Authors’ information

The responsibility for the content of this publication lies with the ICF Research Branch.
